# Ghrelin Attenuates Intestinal Barrier Dysfunction Following Intracerebral Hemorrhage in Mice

**DOI:** 10.3390/ijms17122032

**Published:** 2016-12-06

**Authors:** Yijun Cheng, Yongxu Wei, Wenlei Yang, Yu Cai, Bin Chen, Guoyuan Yang, Hanbing Shang, Weiguo Zhao

**Affiliations:** 1 Department of Neurosurgery, Rui Jin Hospital, Shanghai Jiao Tong University School of Medicine, Shanghai 200025, China; jesny1988@163.com (Y.C.); weiyongxu@icloud.com (Y.W.); yangwenlei@hotmail.com (W.Y.); caiyu10746@sina.com (Y.C.); chenbinns@sjtu.edu.cn (B.C.); hjshb@126.com (H.S.); 2 Department of Neurology, Rui Jin Hospital, Shanghai Jiao Tong University School of Medicine, Shanghai 200025, China; yangguoyuan_neuro@163.com; 3 Neuroscience and Neuroengineering Research Center, Med-X Research Institute, Shanghai Jiao Tong University, Shanghai 200030, China

**Keywords:** intracerebral hemorrhage, ghrelin, intestinal barrier dysfunction, mucosa, intestinal permeability, tight junction, ICAM-1

## Abstract

Intestinal barrier dysfunction remains a critical problem in patients with intracerebral hemorrhage (ICH) and is associated with poor prognosis. Ghrelin, a brain-gut peptide, has been shown to exert protection in animal models of gastrointestinal injury. However, the effect of ghrelin on intestinal barrier dysfunction post-ICH and its possible underlying mechanisms are still unknown. This study was designed to investigate whether ghrelin administration attenuates intestinal barrier dysfunction in experimental ICH using an intrastriatal autologous blood infusion mouse model. Our data showed that treatment with ghrelin markedly attenuated intestinal mucosal injury at both histomorphometric and ultrastructural levels post-ICH. Ghrelin reduced ICH-induced intestinal permeability according to fluorescein isothiocyanate conjugated-dextran (FITC-D) and Evans blue extravasation assays. Concomitantly, the intestinal tight junction-related protein markers, Zonula occludens-1 (ZO-1) and claudin-5 were upregulated by ghrelin post-ICH. Additionally, ghrelin reduced intestinal intercellular adhesion molecule-1 (ICAM-1) expression at the mRNA and protein levels following ICH. Furthermore, ghrelin suppressed the translocation of intestinal endotoxin post-ICH. These changes were accompanied by improved survival rates and an attenuation of body weight loss post-ICH. In conclusion, our results suggest that ghrelin reduced intestinal barrier dysfunction, thereby reducing mortality and weight loss, indicating that ghrelin is a potential therapeutic agent in ICH-induced intestinal barrier dysfunction therapy.

## 1. Introduction

Intracerebral hemorrhage (ICH) is the most devastating subtype of stroke and is associated with high morbidity and mortality. This hemorrhagic disorder accounts for 10%–15% of all strokes, with approximately 2 million cases per year worldwide [1]. Importantly, ICH may not only cause primary damage to the brain itself but also lead to secondary damage to remote organs, such as to the gastrointestinal tract, lung, and heart. Notably, intestinal barrier dysfunction is a common complication after ICH that leads to malabsorption, malnutrition, hypoimmunity, and poor prognosis in patients [2,3].

Intestinal barrier dysfunction is mainly characterized by mucosal injury and increased intestinal permeability [4,5]. The intestines are the core and initiating organs of multiple organ dysfunction under various stressed conditions. The increased permeability of injured intestinal mucosa could result in the translocation of intestinal endotoxin, which, in turn, triggers both a systemic inflammatory response syndrome (SIRS) and a multiple organ dysfunction syndrome (MODS). In the clinic, patients with ICH manifesting gastrointestinal dysfunction have a longer hospitalization period and a higher mortality [2,6]. Although very common, effective strategies for preventing intestinal barrier dysfunction following ICH are still lacking [7].

Ghrelin, a 28-amino-acid brain-gut peptide mainly secreted from the stomach, acts as an endogenous ligand for the growth hormone secretagogue receptor (GHS-R) [8,9]. Ghrelin has numerous biological actions in physiological processes, such as roles in feeding regulation, growth hormone secretion, gastric acid secretion, and metabolism [9,10]. Previous studies have demonstrated that ghrelin can confer protection against intestinal dysfunction in animal models of traumatic brain injury (TBI) [4,5], and sepsis [11]. Nevertheless, until now, no study has addressed the potential effect of ghrelin on ICH-induced intestinal barrier dysfunction. Our study was conducted to test the hypothesis that ghrelin administration attenuates intestinal barrier impairment following ICH in mice.

## 2. Results

### 2.1. Ghrelin Improved Histological Changes in the Intestinal Mucosa after ICH

**Figure 1:**
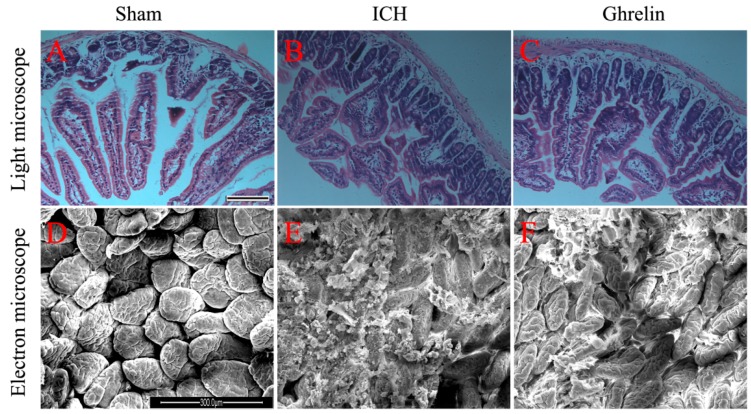
Ghrelin attenuated intestinal mucosal injury after intracerebral hemorrhage (ICH). (**A**) The sham group showed nearly intact mucosa at the histomorphometric level. Scale bar = 100 µm; (**B**) The ICH group presented severe lifting of epithelial cells, thickened and shortened villi, fusion of adjacent villi, and naked lamina propria at the histomorphometric level; (**C**) Less lifting of epithelial cells, thinner and longer villi, less fusion of adjacent villi and naked lamina propria were observed in the ghrelin-treated group at the histomorphometric level; (**D**) The sham group showed smooth-surfaced, orderly arranged villi with few secretions at the ultrastructural level. Scale bar = 300 µm; (**E**) ICH induced marked ultrastructural changes, including increased secretions, irregularly arranged, thickened, sparse, and surface-ruptured villi; and (**F**) Less secretions and more intact villi were observed in the ICH + ghrelin group at the ultrastructural level. *n* = 4 per group.

To investigate the effect of ghrelin administration on the intestinal mucosa post-ICH, ileum sections from each group were observed at histomorphometric and ultrastructural levels. At the histomorphometric level, the samples showed nearly intact mucosa in the sham group (Figure 1A). However, mucosal damage occurred one day post-ICH, indicated by severe lifting of epithelial cells, thickened and shortened villi, fusion of adjacent villi, and naked lamina propria (Figure 1B). After ghrelin administration, the mucosa was protected from injury, presenting less lifting of epithelial cells, thinner and longer villi, less fusion of adjacent villi and fewer naked lamina propria (Figure 1C).

At the ultrastructural level, sections from the sham group showed smooth-surfaced, orderly arranged villi with few secretions (Figure 1D). Following ICH induction, marked ultrastructural changes were observed, including increased secretions, irregularly arranged, thickened, sparse, and surface-ruptured villi (Figure 1E). After treatment with ghrelin, less secretions and more intact villi were observed (Figure 1F).

### 2.2. Ghrelin Attenuated Intestinal Permeability after ICH

**Figure 2:**
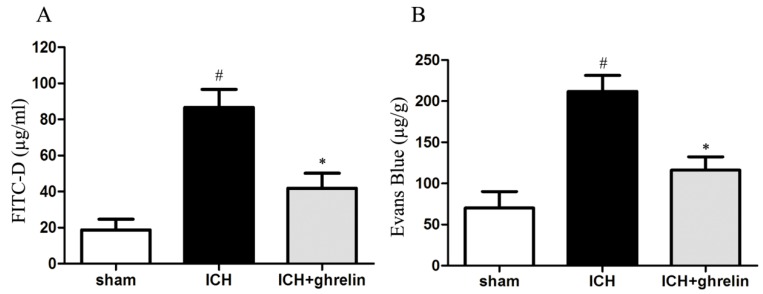
Ghrelin reduced intestinal permeability after ICH. (**A**) The level of fluorescein isothiocyanate conjugated-dextran (FITC-D) was significantly higher after ICH compared to the sham group; however, treatment with ghrelin significantly decreased the FITC-D levels post-ICH; (**B**) Evans blue leakage was markedly increased in the ICH mice compared to the sham mice; ghrelin administration significantly reduced Evans blue extravasation after ICH. The values are the mean ± SD, *n* = 6, # *p* < 0.01 vs. sham group; * *p* < 0.05 vs. ICH group. The data are representative of six independent experiments performed in triplicate.

To evaluate the effect of ghrelin on intestinal permeability post-ICH, fluorescein isothiocyanate conjugated-dextran (FITC-D) and Evans blue extravasation assays were performed. As shown in Figure 2A, low FITC-D level was detected in the sham group. ICH caused a significant increase in the level of serum FITC-D compared to the sham group (*p* < 0.05). However, ghrelin administration markedly decreased FITC-D level post-ICH (*p* < 0.05). Based on Evans blue extravasation, the ICH group exhibited higher dye extravasation compared to the sham group (*p* < 0.05). However, treatment with ghrelin significantly attenuated the leakage of Evans blue caused by ICH (*p* < 0.05; Figure 2B).

### 2.3. Ghrelin Upregulated Tight Junction Protein Expression after ICH

**Figure 3:**
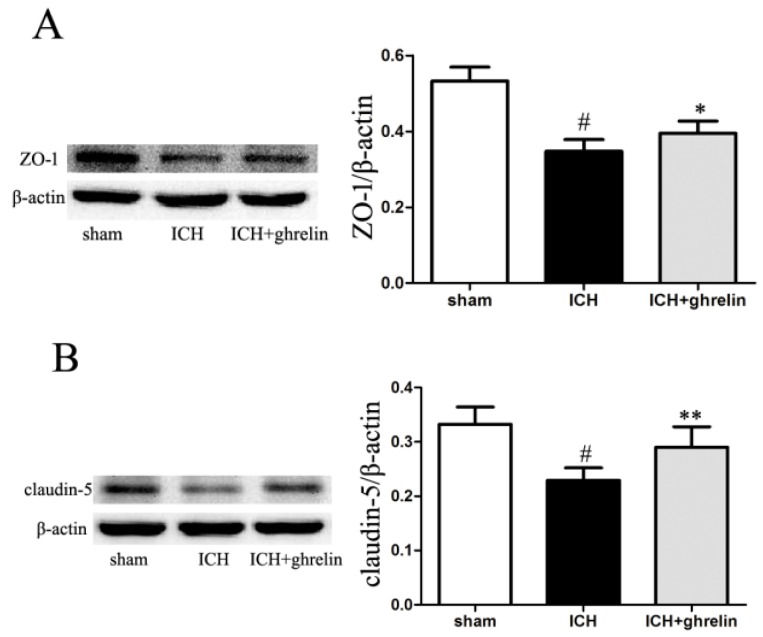
Ghrelin alleviated the disruption of intestinal tight junction after ICH. (**A**) Zonula occludens-1 (ZO-1) protein expression was significantly decreased after ICH compared with the sham group. After ghrelin administration, the ZO-1 protein expression was upregulated post-ICH; (**B**) Claudin-5 protein levels were downregulated in the ICH group compared to the sham group; however, ghrelin increased the protein level of claudin-5 after ICH. The values are the mean ± SD, *n* = 6, # *p* < 0.01 vs. sham group; * *p* < 0.05 vs. ICH group; ** *p* < 0.01 vs. ICH group. The data are representative of six independent experiments performed in triplicate.

Intestinal tight junction disruption post-injury was determined by gap formation and rearrangement [12]. To investigate the tight junction rearrangement, we detected Zonula occludens-1 (ZO-1) and claudin-5 expression based on a Western blot analysis. At the protein level, ZO-1 and claudin-5 expression levels were significantly decreased in the ICH group in compared with the sham group (both *p* < 0.05). However, treatment with ghrelin significantly increased ZO-1 and claudin-5 expression levels post-ICH (both *p* < 0.05; Figure 3A,B).

### 2.4. Ghrelin Downregulated ICAM-1 Expression after ICH

**Figure 4:**
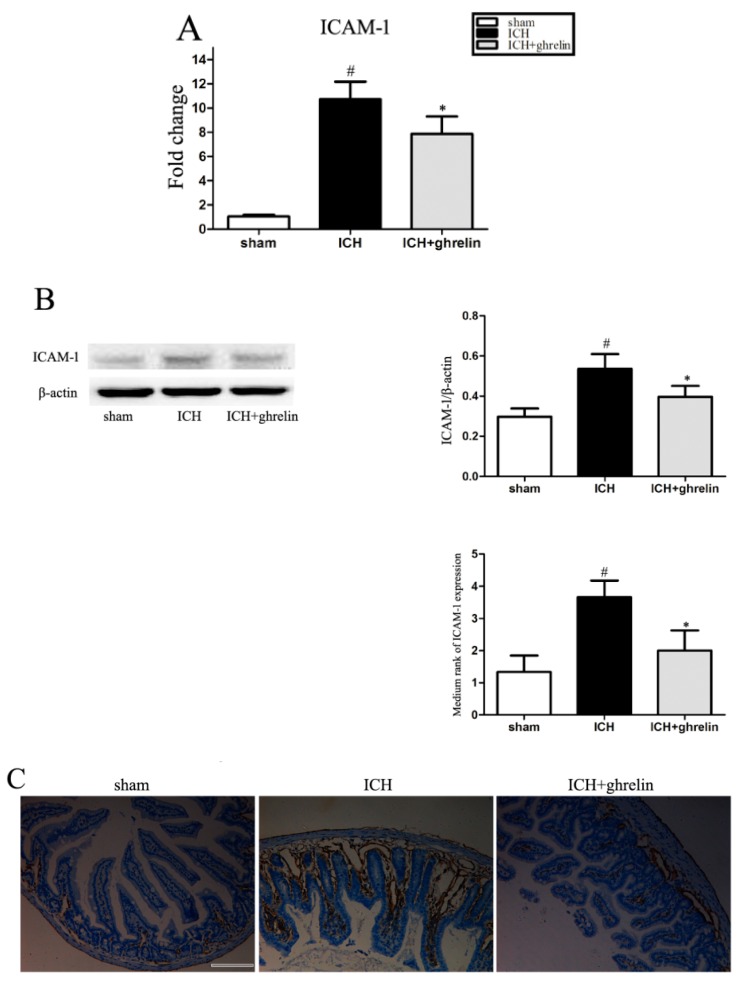
Ghrelin downregulated intestinal intercellular adhesion molecule-1 (ICAM-1) expression after ICH. (**A**) ICAM-1 mRNA was increased following ICH induction compared with the sham group, while ghrelin administration downregulated ICAM-1 expression at the mRNA level post-ICH. *n* = 6; (**B**) ICAM-1 protein levels were higher in the ICH mice than in the sham mice; however, ghrelin significantly inhibited ICH-induced ICAM-1protein levels. *n* = 6; (**C**) Representative photomicrographs of immunostaining for ICAM-1 in each group. ICAM-1 was more clearly observed in the ICH group than that in sham group; however, the ICAM-1-positive cells were significantly reduced in the ghrelin-treated group post-ICH. *n* = 4; Scale bar = 100 µm. The values are the mean ± SD, # *p* < 0.01 vs. sham group; * *p* < 0.05 vs. ICH group; The data are representative of six (**A**,**B**) or four (**C**) independent experiments performed in triplicate.

ICAM-1 contributes to intestinal barrier dysfunction and cytokine release, which is positively correlated with injury severity [13]. To investigate the effect of ghrelin on intestinal ICAM-1 post-ICH, we employed quantitative real-time polymerase chain reaction (RT-PCR), Western blot analysis, and immunohistochemistry to determine changes in ICAM-1 at the mRNA and protein levels. Compared with the sham group, the relative mRNA level of ICAM-1 was markedly increased in the ICH group (*p* < 0.05; Figure 4A). Consistent with the mRNA results, there was a significant increase in ICAM-1 expression at the protein level in the ICH group compared to the sham group (*p* < 0.05; Figure 4B). However, treatment with ghrelin significantly downregulated the expression of ICAM-1 post-ICH at both the mRNA and protein levels (both *p* < 0.05). Additionally, we performed immunohistochemistry to further determine the changes in ICAM-1 in the intestinal tissue from three groups. As shown in Figure 4C, few ICAM-1-positive cells were observed in the sham group. Compared to the sham group, ICAM-1-positive cells were evident in the ICH group (*p* < 0.05). While ghrelin-treated mice exhibited less ICAM-1-positive cells in the intestinal samples than the ICH mice (*p* < 0.05; Figure 4C)

### 2.5. Ghrelin Suppressed the Translocation of Intestinal Endotoxin after ICH

**Figure 5:**
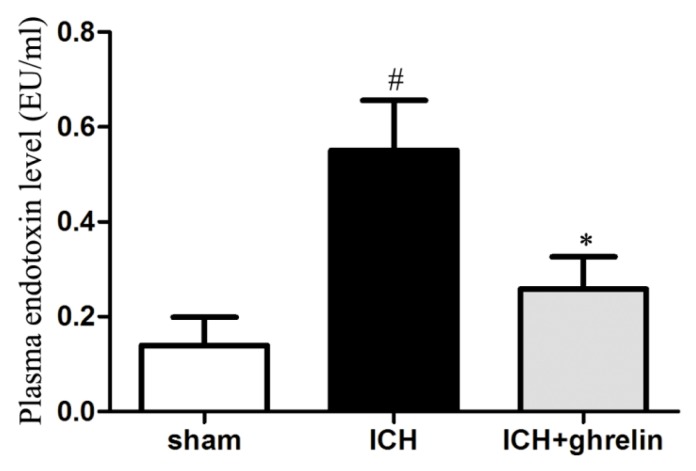
Ghrelin suppressed intestinal endotoxin translocation after ICH. Serum endotoxin levels were significantly higher in the ICH mice than that in the sham mice. After ghrelin administration, serum endotoxin levels were significantly reduced post-ICH. The values are the mean ± SD, *n* = 6, # *p* < 0.01 vs. sham group; * *p* < 0.01 vs. ICH group. The data are representative of six independent experiments performed in triplicate.

To test the effect of ghrelin on intestinal endotoxin translocation post-ICH, we performed a serum endotoxin assay in each group. The results showed that there was a significant increase in serum endotoxin levels in the ICH mice (*p* < 0.05; Figure 5). Treatment with ghrelin significantly reduced the level of endotoxin post-ICH (*p* < 0.05; Figure 5).

### 2.6. Effect of GHrelin on Survival and Body Weight after ICH

**Figure 6:**
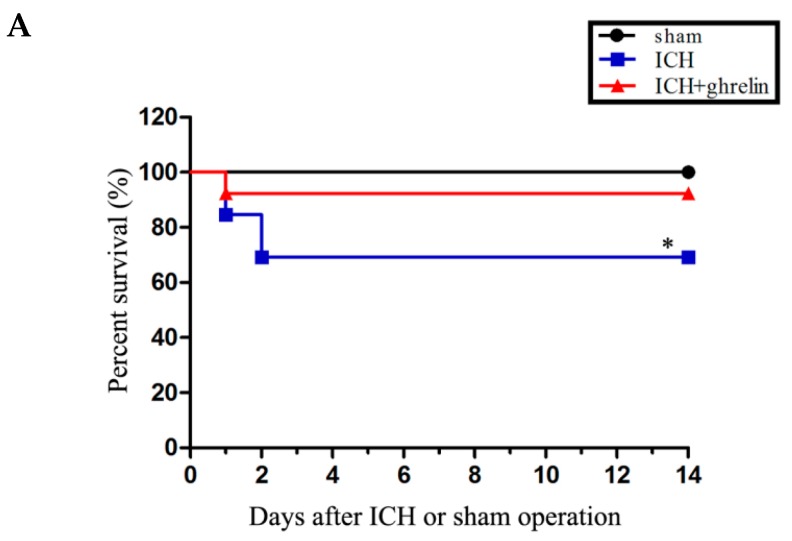

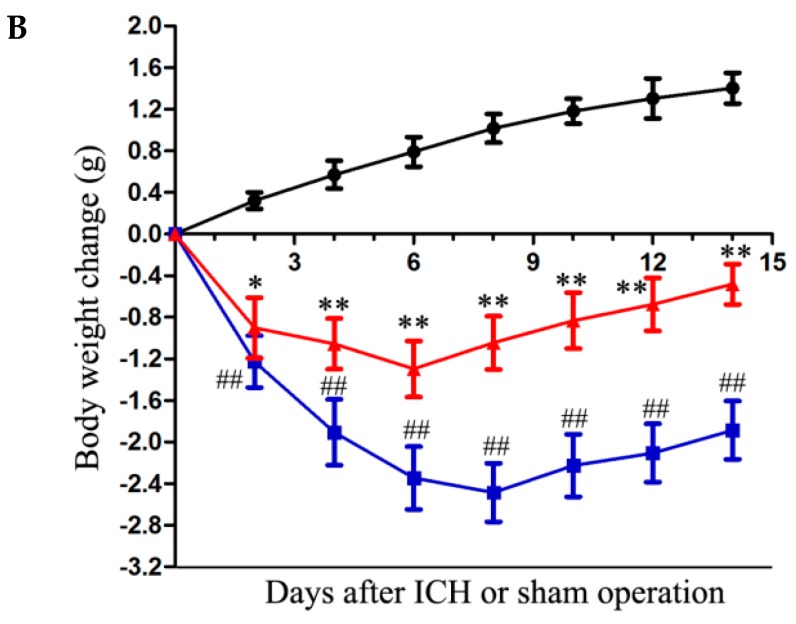
Ghrelin improved the survival rate and reduced body weight loss after ICH. (**A**) The survival rate was reduced following ICH induction without ghrelin administration (ICH, *n* = 26) compared with the sham group (sham, *n* = 13). The administration of ghrelin (ICH + ghrelin, *n* = 26) significantly reduced ICH-induced mortality. There was no significant difference between the sham group and the ICH + ghrelin group; (**B**) The body weight was significantly lower in the ICH mice than that in the sham mice; however, ghrelin significantly attenuated ICH-induced weight loss starting from day 2 to day 14; *n* = 9. The values are the mean ± SD, ## *p* < 0.01 vs. sham group; * *p* < 0.05 vs. ICH group; ** *p* < 0.01 vs. ICH group.

To investigate the effect of ghrelin on survival post-ICH, the Kaplan–Meier method was employed. The number of animals that died of natural causes in each group was recorded during the 14 days. As shown in Figure 6A, the survival rate was 100% in the sham group. Compared with the sham group, the ICH group had a significantly lower survival rate (*p* < 0.05). However, administration of ghrelin markedly decreased the high ICH-induced mortality (*p* < 0.05). Moreover, there was no significant difference in the survival rate between the sham group and the ghrelin-treated group (*p* > 0.05).

To determine the effect of ghrelin on body weight after ICH in mice, changes in body weight were recorded every other day until sacrifice. As shown in Figure 6B, a marked drop in body weight was observed after ICH induction (all *p* < 0.05). After ghrelin administration, weight loss was significantly attenuated starting from day 2 to day 14 post-ICH compared to the ICH group (all *p* < 0.05).

## 3. Discussion

In the current study, we demonstrated that ghrelin attenuated intestinal barrier dysfunction in a murine ICH model. Specially, treatment with ghrelin (i) attenuated ICH-induced intestinal mucosal damage; (ii) reduced FITC-D and Evans blue extravasation post-ICH; (iii) restored the expression of tight junction molecules ZO-1 and claudin-5; (iv) downregulated intestinal ICAM-1 expression; (v) suppressed translocation of intestinal endotoxin; and (vi) improved the survival ratio and alleviated body weight loss caused by ICH.

ICH is a critical brain event that is known as a severely pathological stress. Following ICH, not only the general stress state caused by ICH, but also the brain–gut axis and hypothalamic–pituitary–adrenal axis play an important role in the disruption of intestinal mucosal integrity and barrier function [14]. Intact intestinal mucosa is indispensable for the function of the gut barrier. Prior studies have demonstrated that brain insults may compromise the integrity of the mucosal structure, leading to stress ulcer and gastrointestinal bleeding depending on the severity of the injury [3]. Several animal models of neurologic diseases, including TBI [4,5,14,15,16], subarachnoid hemorrhage (SAH) [17], and ischemic stroke [18], have been proven to cause damage to the intestinal mucosa. However, the changes in the intestinal mucosal structure post-ICH have not been systematically studied in vivo. In this study, we observed alternations in the intestinal mucosa after ICH at both histomorphometric and ultrastructural levels. At the histomorphometric level, severe lifting of epithelial cells, thickened and shortened villi, fusion of adjacent villi, and naked lamina propria were observed after ICH. At the ultrastructural level, increased secretions, irregularly arranged, thickened, sparse, and surface-ruptured villi were observed following ICH. However, this phenomenon was significantly inhibited by treatment with ghrelin, whose concentrations in the serum could be increased by several hundred-fold as early as 1 min after exogenous ghrelin administration compared to the normal endogenous ghrelin concentrations [19]. These findings, in combination with those of Bansal et al. [5], suggest that ghrelin administration attenuated ICH-induced intestinal mucosal injury.

Intestinal permeability directly reflects the function of the intestinal barrier [20,21]. FITC-D and Evans blue are non-absorbed macromolecules that are used as indicators of intestinal permeability [22]. In our study, we found that treatment with ghrelin significantly attenuated FITC-D and Evans blue leakage following ICH, thus reducing the intestinal permeability. The results are in line with prior studies [5]. As is known, one of the key characteristics of gut barrier dysfunction is increased levels of peripheral blood endotoxin [23], which is also an indirect indicator of intestinal permeability [24]. Endotoxin is a component of the outer cell membrane of Gram-negative bacteria. Increased intestinal permeability caused by various stress insults permits intestinal endotoxin translocation into the blood circulation, triggering systemic inflammatory response and the release of various inflammatory mediators [15,25]. These mediators might further exacerbate sepsis, SIRS and MODS. Notably, ICH induction results in blood–brain barrier breakdown and increases its permeability. Blood endotoxin might cross the blood–brain barrier and exacerbate neuroinflammation in the injured brain, in turn worsening the prognosis of patients [26,27]. In the present study, serum endotoxin was increased after ICH and reversed by ghrelin administration. Collectively, these data suggest that ghrelin could reduce intestinal permeability and subsequently suppress endotoxin translocation, preserving the intestinal barrier function following ICH.

In the intestine, the epithelial cell-to-cell contact, strengthened by tight junctions, defines the barrier function [28]. Tight junctions are composed of different molecules, including claudins [29], occludin [30], ZOs [31,32], tricellulin [33], myosin light chain kinase (MLCK) [34], and junctional adhesion molecule (JAM) [35]. Various injuries can contribute to a reduction of tight junction proteins, such as claudin-5 and ZO-1 [36]. In this study, ghrelin administration markedly increased ICH-induced downregulation of tight junction molecules, claudin-5 and ZO-1 at the protein level, suggesting that ghrelin plays a vital role in the maintenance of intestinal barrier function.

ICAM-1, an adhesion molecule, has been shown to participate in cytoskeletal and junctional reorganization in epithelial cells, mediating firm adhesion [13]. Inflammatory mediators, such as interleukin (IL)-1β, tumor necrosis factor (TNF)-α, Interferon (IFN)-γ, and IL-17 mainly account for the production of ICAM-1 [37]. Ultrastructurally, ICAM-1 is found in the extracellular matrix and in the endoplasmic reticulum, suggesting secretion, shedding, or release during cell lysis [38]. Previous studies have shown that acute intestinal injury caused by several neurological diseases, including traumatic brain injury [16,39] and subarachnoid hemorrhage [40], upregulates ICAM-1 expression. Notably, the inhibition of ICAM-1 may significantly attenuate intestinal barrier dysfunction [41,42,43]. Moreover, ICAM-1 has been proven to be responsible for the upregulation of MLCK, which is closely involved in intestinal barrier dysfunction [13]. However, the relationship between ghrelin and ICAM-1 remains controversial. Kellokoski et al. [44] found that ghrelin administration increased ICAM-1 expression in endothelial cells, further enhancing monocyte adhesion. On the other hand, Zhang et al. [45] found that ghrelin may inhibit ICAM-1 expression and that a gene knockout of ghrelin receptor enhanced ICAM-1 expression. The phenomenon is likely related to the different cell models considered. In this study, we found the intestinal ICAM-1 gene expression was increased after ICH. After ghrelin administration, ICAM-1 was significantly decreased post-ICH. These findings suggest that ghrelin could serve as an ICAM-1 inhibitor, further alleviating intestinal barrier dysfunction following ICH.

The function of the intestinal barrier is closely associated with body weight preservation at the whole-body level. In our study, ICH mice exhibited a marked drop in body weight from two to 14 days compared with sham mice; however, treatment with ghrelin significantly attenuated the weight loss caused by ICH, indicating ghrelin could induce weight gain and better nutritional status in mice [46,47]. Nutritional status has a substantial impact on the immunity and prognosis of brain injury patients. Aside from attenuating the intestinal barrier dysfunction as mentioned above, ghrelin might also protect the intestine by multiple physiological functions, including stimulating growth hormone release [8], regulating insulin [48], stimulating appetite [49], and modulating energy metabolism [50]. However, the impact of ghrelin administration on growth hormone, insulin, appetite, and metabolism in ICH requires further investigation. Recently, ghrelin has been documented to exert neuroprotection in several models of neurologic diseases, including ischemic stroke [51], TBI [47], and SAH [52]. More interestingly, studies on the effects of ghrelin on ischemic stroke [51] and TBI [47] indicated that ghrelin-mediated alleviation of brain injury contributed to the prevention of weight loss after brain insults. Therefore, we speculate that in addition to the direct protective effects, the beneficial effects of ghrelin on intestinal barrier dysfunction and weight loss after ICH may also relate to ghrelin-mediated alleviation of brain injury. However, whether this is true for ICH still needs further study.

## 4. Experimental Section

### 4.1. Experimental Groups and Drugs

Male 6- to 8-week-old C57BL/6 mice were obtained commercially from the Experimental Animal Center of the Chinese Academy of Sciences, Shanghai, China. Animals were randomly divided into the following three groups: (i) sham group (*n* = 18); (ii) ICH group (*n* = 50); and (iii) ICH + ghrelin group (*n* = 50). All animal procedures were approved by the Med-X Research Institute, Shanghai Jiao Tong University (SYXK2013-0034) and were performed in accordance with the rules of the US National Institutes of Health Guidelines and The Guidelines on the Humane Treatment of Laboratory Animals (MOST 2006a) established by China. Mice were housed at a constant room temperature and humidity under a 12/12 h light/dark cycle and specific pathogen-free (SPF) conditions including sterilized food and water. All efforts were made to reduce the number of mice used and to minimize suffering. On day 1 post-ICH, animals were sacrificed, and the small intestinal samples were harvested for further study.

In the ghrelin-treated group, ghrelin (Tocris Bioscience, Ellisville, MO, USA) was dissolved with physiological saline and injected intraperitoneally (IP) at 0 (10 μg) and 1 h (10 μg) following ICH induction. The dosing and delivery route were performed according to previous studies [53,54,55,56]. Evans Blue dye was purchased from Sangon (Sangon Bio, Shanghai, China); all other drugs used in this study were purchased from Sigma (St. Louis, MO, USA).

### 4.2. ICH Model

The ICH model was induced by stereotactically injecting autologous blood, as previously described with minor modification [57]. Briefly, mice were anesthetized with an intraperitoneal injection of ketamine/xylazine (100/10 mg/kg) and placed prone onto a stereotactic frame (RWD Life Science Co., Shenzhen, China). Then, a 1 mm burr hole was drilled 2.3 mm lateral to the midline and 0.2 mm anterior to bregma. A volume of 25 μL autologous blood was collected from a tail cut by a surface-heparinized Hamilton syringe (Hamilton, Reno, NV, USA). A needle was deeply advanced 3.0 mm into the right striatum and the autologous blood was injected in two stages using a microinfusion pump (WPI, Sarasota, FL, USA). First, 5 μL autologous blood was injected at a rate of 2 μL/min. Following a 7 min interval without injection, the remaining 20 μL autologous blood was delivered at the same rate. After injecting, the needle was left in place for 10 min to prevent blood backflow. Subsequently, the drilled hole was sealed with bone wax, and the skin was sutured. Sham controls had only a needle insertion.

### 4.3. Histopathology

Damage to the small intestinal mucosa was assessed by histomorphometric and ultrastructural examinations. For histomorphometric studies, ileum segments were harvested from mice in each group at 1 day and then fixed in 4% paraformaldehyde. Samples were embedded in paraffin and cut into 4 μm pieces, mounted on glass slides, and then stained with hematoxylin and eosin (H&E, Beyotime Biotechnology, Haimen, China). Histopathological changes of the samples were observed by a blinded observer.

For ultrastructural studies, briefly, fresh ileal samples were harvested on day 1 in three groups and double fixed in 2% glutaraldehyde and 1% osmic acid. After washing, the samples were dehydrated in increasing concentrations of ethanol, dried to the critical point, and then sputter coated with BAL-TEC ions. Samples were scanned using a QUANTA-200 scanning electron microscope (Philips, Amsterdam, The Netherlands). Ultrastructural changes of the samples were observed following a blinded procedure.

### 4.4. Intestinal Permeability

Intestinal permeability at day 1 was determined using fluorescein isothiocyanate conjugated-dextran (FITC-D, 4 kDa) and Evans blue as previously described [58,59,60]. For FITC-D assays, 6 h before sacrifice, the animals underwent laparotomy under deep anesthesia and a 5 cm segment of the ileal loop was ligated beginning at 3 cm proximal to the ileocecal junction. Then, previously prepared FITC-D solution (200 µL of PBS containing 25 mg/mL FITC-D) was gently injected into the ileal sac, and the abdomen was sutured with silk stitches. After 30 min, blood samples (50 µL) were collected by cardiac puncture and centrifuged at 6000× *g* for 5 min at 4 °C. FITC-D concentrations were determined using a fluorescence spectrophotometer (Thermo Scientific, Wilmington, DE, USA) at an excitation wavelength of 485 nm and an emission wavelength of 515 nm. A standard curve was established to calculate the serum FITC-D concentration.

For Evans blue assessments, a laparotomy was performed under anesthesia and intestinal sacs were prepared as described previously [60]. The luminal contents were washed out gently with PBS. Then, previously prepared Evans blue solution (200 µL of 1.5% (weight-to-volume, *w*/*v*) Evans blue in PBS) was gently injected into the lumen. The intestinal sacs were immediately incubated in Krebs buffer (20 mL) at 95% O_2_ at 37 °C for 30 min. After washing three times, the sacs were dried at 37 °C for 24 h, weighted, and then incubated with formamide (1 mL) at 50 °C for another 24 h. The absorbance was measured using a spectrophotometer at a wavelength of 610 nm. A standard curve was used to quantify the content of Evans blue in the formamide.

### 4.5. Serum Endotoxin Level

Blood samples were drawn from the hearts of the mice prior to sacrifice and then centrifuged at 6000× *g* for 5 min at 4 °C. The serum levels of endotoxin content on day 1 were assayed using achromogenic substrate limulus amebocyte lysate (LAL) kit (TAL, Xiamen, China) according to the manufacturer’s protocol. The results were expressed as EU/mL.

### 4.6. Quantitative RT-PCR

Total RNA was extracted from terminal ileums using Trizol Reagents (Invitrogen, Carlsbad, CA, USA) according to the manufacturer’s instructions. Before RNA was reversely transcripted to cDNA with a Prime Script RT reagent kit (TaKaRa, Otsu, Japan), the RNA quantity was determined via a spectrophotometric analysis (OD_260/280_). Primers were synthesized and used to amplify the target genes as follows: ICAM-1: 5′-GCCTCCGGACTTTCGATCTT-3′ (forward), 5′-GTAGACTGTTAAGGTCCTCTGCGT-3′ (reverse); β-actin: 5′-GTGACGTTGACATCCGTAAAGA-3′ (forward), 5′-GCCGGACTCATCGTACTCC-3′ (reverse). RT-PCR was performed using an SYBR Green kit (TaKaRa, Otsu, Japan). All results were calculated by using the Δ*C*_t_ method with SDS software (version, Applied Biosystems, Carlsbad, CA, USA).

### 4.7. Western Blot Analysis

Total protein was extracted from the terminal ileums with a mixture of RIPA buffer (Millipore, Bedford, MA, USA). The supernatant was collected via centrifugation at 12,000× *g* for 10 min at 4 °C. Protein concentrations were measured using an enhanced BCA protein assay kit (Thermo, Waltham, MA, USA). Equal amounts of protein (50 µg) were loaded on each lane of a 10% sodium dodecyl sulfate-polyacrylamide gel by electrophoresis (SDS-PAGE) and then electro-transferred to a PVDF membrane (Millipore, Temecula, CA, USA). The membrane was blocked in 5% non-fat milk for 1 h at room temperature and then incubated with primary antibodies against ZO-1 (1:500 dilution), claudin-5 (1:400 dilution, both from Cell Signaling Technology, Beverly, MA, USA), and β-actin (1:10,000 dilution, Abcam, Cambridge, UK) at 4 °C overnight. After washing, the membrane was incubated with HRP-labeled secondary antibody (1:5000 dilution, Hua An, Hangzhou, China) for 1 h at room temperature. The bands were visualized using ECL chemiluminescence reagent (Pierce, Rockford, IL, USA) and quantified based on the mean pixel density using Quantity One software (Version 4.6.2, Bio-Rad, Hercules, CA, USA).

### 4.8. Immunohistochemistry

The paraffin sections of the ileum tissue were dewaxed in xylene and rehydrated. The sections were incubated in 3% H_2_O_2_ in PBS for 10 min, and blocked in PBS containing 5% normal goat serum for 1 h at room temperature, followed by incubation with the primary antibodies, anti-ICAM-1 (1:100 dilution, Abcam, Cambridge, UK) at 4 °C overnight. After washing, the sections were developed with the ABC kit and detected by DAB (both from Vector Laboratories, Burlingame, CA, USA). Then, the sections were counterstained with hematoxylin. ICAM-1-positive cells were identified and photographed by an investigator blinded to the group identities. The sections were evaluated by assessing the intensity of the staining (five grades) as previously described [16]. “0” indicates no detectable positive cell; “1” indicates very low density of positive cells; ‘‘2” indicates a moderate density of positive cells; ‘‘3” indicates the higher, but not maximal density of positive cells; and ‘‘4” indicates the highest density of positive cells.

### 4.9. Statistical Analysis

The analysis was performed using SPSS 16.0 (SPSS Inc, Chicago, IL, USA). Differences between multiple groups were analyzed by one-way ANOVA followed by the Student–Newman–Keuls method. The survival rate was determined by the Kaplan–Meier method followed by the log-rank test. Quantitative data were expressed as the mean ± SD. *p* < 0.05 was considered to indicate a statistically significant difference.

## 5. Conclusions

In summary, the present study suggests that ghrelin attenuates ICH-induced intestinal barrier dysfunction by alleviating intestinal mucosal injury, reducing intestinal permeability, increasing tight junction molecules, inhibiting ICAM-1 expression, and suppressing endotoxin translocation. Further studies using ghrelin knockout and ghrelin receptor mice are required to examine the exact role of ghrelin in the intestinal protection of ICH animal models. Additionally, the effect of ghrelin on the secondary brain injury caused by ICH should be investigated. This knowledge is necessary before a role of ghrelin in the systemic treatment of ICH can be envisaged.
